# Measuring the drafting alignment of patent documents using text mining

**DOI:** 10.1371/journal.pone.0234618

**Published:** 2020-07-10

**Authors:** Davit Khachatryan, Brigitte Muehlmann

**Affiliations:** 1 Division of Mathematics and Science, Babson College, Babson Park, Massachusetts, United States of America; 2 Division of Accounting and Law, Babson College, Babson Park, Massachusetts, United States of America; University of California Los Angeles, UNITED STATES

## Abstract

How would an inventor, entrepreneur, investor, or patent examiner quantify the extent to which the inventive claims listed in a patent document align with patent specification? Since a specification that is poorly aligned with the inventive claims can render an invention unpatentable and can invalidate an already issued patent, an effective measure of alignment is necessary. We define a novel measure of drafting alignment using Latent Dirichlet Allocation (LDA). The measure is defined for each patent document by first identifying the latent topics underlying the claims and the specification, and then using the Hellinger distance to find the proximity between the topical coverages. We demonstrate the use of the novel measure for data processing patent documents related to cybersecurity. The properties of the proposed measure are further investigated using exploratory data analysis, and it is shown that generally alignment is positively associated with the prior patenting efforts as well as the tendency to include figures in a document.

## Introduction

The receipt of the patent application at the patenting office kicks off the patent prosecution, which is the process as part of which one or more patent examiners attempt to determine the patentability of the invention outlined in the application. If the invention is of patentable subject matter, then its novelty, non-obviousness, and usefulness are necessary (but not sufficient) conditions for patentability, according to the title 35 of the United States Code (USC). In addition, and as is the focus of this article–the inventive content listed as part of a document’s claims should be in line with the description of the invention that appears as part of patent specification. Indeed, the Manual of Patent Examining Procedure (MPEP) states that “The contents of an application, to be complete, must include a specification containing a written description of the invention … The example(s) and description should be of sufficient scope as to justify the scope of the claims.” [1]. In addition, according to the §112(a) of Title 35 of USC, “The specification shall contain a written description of the invention, and of the manner and process of making and using it, in such full, clear, concise, and exact terms as to enable any person skilled in the art to which it pertains, or with which it is most nearly connected, to make and use the same, and shall set forth the best mode contemplated by the inventor or joint inventor of carrying out the invention.” [2] The latter quoted statement is often referred to as the “written description” requirement.

The lack of alignment between claims and specification can result in patent rejection during the patent prosecution. In particular, if the submitted patent application fails to describe the invention in a manner that satisfies the written description requirement, then the patent cannot be granted in the current form. This unnecessarily prolongs the patent prosecution phase, and may ultimately end in rejection.

In addition to patent applications, issues may ensue for already granted patents, because granted patents can be challenged for validity throughout their lives, which may result in millions of dollars of litigation costs. A number of researchers have documented the tendency for low-quality patents due to inefficient prosecuting practices [3–6]. [3] note that due to time and information constraints faced by patent examiners “… it is hardly a surprise that the PTO makes mistakes during the initial process of patent review, granting patents that, on the merits, should never have been issued.” [3] Mann and Underweiser [7], studying patent post-grant validity using patents considered for validity by the Federal Circuit Court of Appeals, note that “More than a quarter of the patents held invalid in the data set suffered from drafting problems, which generally reflect a failure to include a specification that adequately describes and enables an invention that is delineated with definiteness in the claims.” [7]

The observations emphasized above imply the importance of a tool that can be used to measure alignment between claims and specification of a document. Such a tool would help inventors, entrepreneurs, investors and patent examiners. The purpose of the current work is to propose a novel measure of alignment, that from hereon will be referred to as *drafting alignment*.

## Brief background on patenting in the U.S.

During patent examination or, in the patenting parlance “prosecution,” examiners verify that the invention is of patentable subject matter and that it is adequately described in the (“non-provisional”) patent application. Moreover, examiners try to establish or refute the novelty, usefulness, and non-obviousness of the invention [8], [9], [2]. The patent is granted if all of these criteria are met. A granted patent gives its owner a temporary (generally for 20 years) monopoly to exercise a right to “exclude others from making, using, offering for sale, or selling the invention throughout the United States or importing the invention into the United States …” [10].

The heart of the document in terms of what the inventor considers as the novelty of the invention is outlined in the document’s claims section [11]. Claims can be either independent or dependent. While independent claims outline specific features of the invention and are “standalone” since they are not based on other claims, the dependent claims rest on independent claims of the same document and further narrow them [2].

The claims section is usually at the end of the document, preceded, among other sections, by the specification of the patent. The specification is the part of the document where a comprehensive description of the invention is presented and often titled as “Detailed Description of the Invention,” “Detailed Description of the Preferred Embodiments,” or through other similar titles. It is the purpose of the specification section to describe the invention in sufficient detail. As the specification should justify the scope of the claims, examiners scrutinize the claims of the document looking for necessary justification described in the specification during the patent prosecution. Additionally, for the purposes of verifying the novelty of the invention examiners search the already existing inventive knowledge (the “prior art”). There are a few classification systems that either were used or are currently being used to ease the search for prior art.

The United States Patent Classification (USPC) system is a framework developed by the USPTO that facilitated the search for prior art among the existing patents. It is a classification system, organized in classes and sub-classes, based on the presented subject matter [12]. Effective January 1, 2015 the USPC was substituted by the Cooperative Patent Classification (CPC) system. CPC is used in both the USPTO and the European Patent Office. It is based on the International Patent Classification (IPC) system, a system administered by the World Intellectual Property Organization (WIPO). With both IPC and CPC, technology is broken down into eight sections. The hierarchical organization of IPC/CPC codes is based on sections, within which are classes, further broken down into subclasses, then main groups, and finally subgroups. As an example, in a section-class-subclass-main group combination of *G06F21* the section identifier G stands for *Physics*. Further, Class 06 represents subject matter related to *Computing*, *Calculating*, *Counting*, Subclass F pertains to *Electric Digital Data Processing*, and finally Main Group 21 uniquely identifies the subject matter of the entire combination, namely: *Security arrangements for protecting computers*, *components thereof*, *programs or data against unauthorised activity*. Main group 21 is further broken down into Subgroups. For example, within Main Group 21, Subgroup 31 corresponds to *User Authentication* while Subgroup 44 stands for *Program or Device Authentication*. While the IPC classification ends at the Subgroup level, CPC adds further granularity. Of note, despite the fact that the key structural components of IPC and CPC systems are the same, the class numbers assigned to an invention may vary.

It is important to note that according to the American Inventor Protection Act (AIPA) of 1999, patent applications are made available to the public after 1.5 years from the submission regardless of their granting status. For example, if a patent application is not granted, because it does not satisfy the written description requirement, it is still made available to the public 1.5 years after submission. Also, if a patent is granted but subsequently invalidated, then the document remains in the public domain. What one ends up with is the public disclosure of inventive knowledge without any patent rights to exclude others from utilizing the invention. Knowing how to measure alignment between claims and the specification, and thus safeguard against submitting to the USPTO a poorly aligned application, can be crucial to the applicant for avoiding such pitfalls.

## A novel measure of drafting alignment

The measure that will be defined in this section will use as building blocks a previous attempt in defining alignment [7], as well as a common topic modeling approach, the LDA [13]. Each of these building blocks is discussed in the remainder of this section, followed by the formal definition of the proposed measure.

### Post-grant validity and drafting alignment

To understand patent quality, [7] study all patents for which the legal validity was considered by the Federal Circuit since 2003. In particular, the authors analyze the association that various factors pertaining to the invention, application, and prosecution may have with the likelihood of the patent staying valid after being granted. The authors model the probability of a patent remaining valid post-grant using logistic regression. Among predictors is the degree of alignment between patent claims and specification, which the authors refer to as “drafting quality.” According to their work, the odds of a patent staying valid post-grant increased, the closer the claims are aligned with the specification, ceteris paribus.

The measure defined by the authors deserves a note. For each patent, after pre-processing both the claims and the specification, each of these bodies of text is mapped to a vector. The vector is constructed in a way that if a word appears in the corresponding text (e.g., the claims section) then the entry in the vector corresponding to that word will be one, otherwise zero. Accordingly, each patent is mapped to a pair of vectors: one vector corresponding to claims and another vector to specification. Having arrived at these pairs of vectors, for each document the authors used the Euclidean distance between the corresponding two vectors to measure the alignment. A patent for which claims are poorly aligned with the specification will score high on the defined metric, and conversely, low scores will correspond to well-aligned patents.

Two observations regarding the measure used by [7] need to be made. Our first observation is related to the binary encoding. As has been noted by the authors themselves, their measure does not account for the frequency of words in each text, but only accounts for whether or not a word appears in the corresponding text. One could argue that not only the mere presence of a concept in both claims and specification sections should be taken into account, but also how often that concept appears in each corresponding text. If a concept is given major attention in claims (e.g. by frequently using it), but is mentioned only sparingly in the specification–that could be indicative of a lack of description or justification in the specification to support what is said in claims. The second observation pertains to the very use of words for comparing the two bodies of text (i.e. claims and specification sections). The authors’ use of single words (unigrams) in the definition is arbitrary. The question arises as to why the claims (and the specification) are not instead tokenized to constructs such as bigrams, trigrams, or any given number of words that are either consecutive or separated by a window of fixed length. On the other hand, regardless of the tokenization, the very fact that the degree of similarity of the two texts would be based on matching single words (or combinations) across texts seems limiting.

Due to the aforementioned observations, the identification of topics (rather than marking words) constituting each body of text, and the comparison of the topical coverages (rather than occurrences of words) across the claims and specification emerges as a meaningful alternative for the definition of drafting quality. Our measure of drafting alignment is developed along those lines. It is motivated by the important work of [7], but is more robust in that it effectively addresses the above-mentioned limitations. Its cornerstone is LDA, which is a popular approach for identifying latent topics in text, and which is described in the following subsection.

### Latent Dirichlet Allocation (LDA)

Identification of topics making up text corpora is an important part of the analysis of text data. Knowledge of the building blocks of given text can be useful when trying to gain an understanding of the themes present in the text. Having an analytic method to replace the otherwise tedious visual scanning or skimming of text can help the researcher or practitioner streamline the process of data exploration, save time, and reduce subjectivity. LDA is one such method that has gained significant popularity in recent years.

LDA is a statistical model developed by Blei, Ng, and Jordan in 2003 [13]. Assume the existence of *T* latent topics that are responsible for the observed *D* documents constituting the corpus at hand. Also, assume that each latent topic is presented as a probability distribution over a given vocabulary *W* = (*w*_1_,*w*_2_,…,*w*_*n*_) where each *w*_*i*_ is a (unique) word present in the given corpus. As a hypothetical example, a topic such as “cryptography” could be represented as a distribution assigning relatively high probabilities to words such as encryption, cipher, and key; while a topic “machine learning” would be represented by a distribution that assigns relatively high probabilities to words such as regression, validation, and Bayes. Further, assume that each word is generated according to a topic. Each document is produced by choosing a distribution over topics, and once that distribution is chosen, then each word in that document is generated by first randomly choosing a topic, and then a word from that topic [13]. Note that we used the term “word” in this description although *w*_*i*_ can be, and often is, some lexical transformation of a word such as a stem.

More technically, assume a corpus consisting of *D* documents based on the vocabulary *W* made up of *n* unique words. Assume that the probability of “seeing” the *i*^th^ word in a given *d* document is given by the following mixture distribution [13], [14]:

$$\begin{array}{l} p\left(w_{i}|\theta_{d}\right)\\ =\sum_{j=1}^{T}\theta_{j}(d)p\left(w_{i}|z_{i}=j,\Phi \right) \end{array}$$

where $\theta_{j^{\left(d\right)}}$ is the probability of *j*^th^ topic’s occurrence as part of the document under consideration. Moreover, *z*_*i*_ denotes the latent topic from which the word *w*_*i*_ comes. Statistically, for each latent topic *z* there is a corresponding multinomial distribution over the vocabulary *W* with word occurrence probabilities given by $p(w|z=j,\Phi )=\phi_{w}^{\left(j\right)}$ and organized into an unknown *T*×*n* parameter matrix **Φ**. Note that for any given *j* the distribution *p*(*w*|*z* = *j*,**Φ**) can be thought of as the “definition” of the *j*^th^ latent topic, and it does not vary from document to document. On the other hand $\theta_{j^{\left(d\right)}}$, the contribution of *j*^th^ topic in document *d*, is document-specific.

A characteristic feature of LDA is that for each document *d*, the *T*-dimensional random vector of mixing proportions $\theta_{d}=(\theta_{1^{\left(d\right)}},\theta_{2^{\left(d\right)}},\ldots ,\theta_{T^{\left(d\right)}})$ is assumed to be having a Dirichlet prior that is based on a corpus-specific (hyper)parameter vector **α** = (*α*_1_,*α*_2_,…,*α*_*T*_) with *α*_*j*_>0. The probability density function for the Dirichlet prior is given below, where $\Gamma (x)=\int_{0}^{\infty }u^{x-1}e^{-u}du$ is the Gamma function:

$$p(\theta_{d}|\alpha )=\frac{\Gamma (\sum_{j=1}^{T}\alpha_{j})}{\prod_{j=1}^{T}\Gamma (\alpha_{j})}\prod_{j=1}^{T}\theta_{j^{\left(d\right)}}^{\alpha_{j}-1}$$

The estimation problem in LDA is that of finding the posterior probability distribution of the latent variables given the document *d*:

$$p(\theta_{d},z|w_{d},\alpha ,\Phi )=\frac{p(\theta_{d},z,w_{d}|\alpha ,\Phi )}{p(w_{d}|\alpha ,\Phi )}$$

where **z** is the random vector of latent topics, $w_{d}=(w_{1},w_{2},\ldots ,w_{n_{d}})$ represents the words in the document *d*, and *p*(**w**_d_|**α**,**Φ**) is the marginal distribution of the document under consideration given by:

$$p(w_{d}|\alpha ,\Phi )=\int \int \ldots \int p(\theta_{d}|\alpha )\left(\prod_{i=1}^{n_{d}}\sum_{j}p(z_{i}=j|\theta_{d})p(w_{i}|z_{i}=j,\Phi )\right)d\theta_{1^{\left(d\right)}}d\theta_{2^{\left(d\right)}}\ldots d\theta_{T^{\left(d\right)}}$$

Due to its intractability, the posterior is only approximated often using approaches such as variational inference [15], [13], Markov Chain Monte Carlo methods [16], [15], and expectation-propagation [17], among other approaches.

### Definition of drafting alignment

Given a corpus consisting of *D* documents, we define drafting alignment as follows. First, each document is pre-processed by i) removing redundancies such as punctuation, stop words, numerals (Arabic and Roman), and Greek letters; ii) converting the text to lower-case; iii) removing generic patent-related language, such as “embodiment”, “claim”, “prior art”, etc.; iv) stemming each resultant word in each document using Porter stemming [18]; v) tokenizing each document to unigrams yielding the vocabulary *W* of unique stemmed unigrams; and vi) constructing the “document-term” matrix **M** that contains document indices as the rows and unique terms from the vocabulary *W* as the columns. Assuming the existence of *T* latent topics throughout the pre-processed corpus, LDA is applied to uncover the latent topics based on matrix **M**. Note that LDA is fit only once (based on **M**), after which for any given document the most likely topic is assigned to each term in claims and specification, respectively. This assignment is carried out by approximating *p*(*z*_*i*_|**w**_d_) using the variational posterior multinomial parameters $\delta_{i}=({\delta_{i}}_{1},{\delta_{i}}_{2},\ldots ,{\delta_{i}}_{T})$ that are derived during the variational inference [13]. The most likely topic for each term is then chosen as ${argmax}_{j^{\prime }}\delta_{i}=\{j|\forall j^{\prime }\neq j:{\delta_{i}}_{j^{\prime }}<{\delta_{i}}_{j}\}$ [13]. After this assignment, the relative frequency distribution of topics is calculated separately for the claims and specification, resulting in a pair of distributions *P* = (*p*_1_,*p*_2_,…,*p*_*T*_) and *Q* = (*q*_1_,*q*_2_,…,*q*_*T*_), respectively, per each document. Finally, our measure of drafting alignment is defined as one minus the Hellinger distance [19] between the two distributions as shown below.

$$1-\frac{1}{\sqrt{2}}\sqrt{\sum_{i=1}^{T}{\left({\sqrt{p}}_{i}-{\sqrt{q}}_{i}\right)}^{2}}$$

The choice of the Hellinger distance deserves a special note. To measure the discrepancy between the topical distributions in claims and specification we initially considered the possibility of using the Kullback-Leibler divergence [20], which for discrete probability distributions is given by $K(P,Q)=\sum_{x}P(x)log\frac{P(x)}{Q(x)}$ [21]. It was initially expected that the measure could potentially be used to assess the amount by which the topical coverage in the specification diverges from what is stated in claims or, put differently, how accurately the specification “approximated” the contents of the claims. However, we discovered that Kullback-Leibler divergence was not suitable for the data that we had at hand for two reasons. First, because for a number of documents for which *Q*(*x*) = 0 in our data *P*(*x*)≠0 and thus *K*(*P*,*Q*) would be undefined. The second limitation of *K*(*P*,*Q*) is that it is not symmetric and thus does not qualify as a distance, strictly speaking. One way around the asymmetry was to use the Jensen-Shannon divergence instead [22]. That would, however, still leave the first issue unresolved. The Hellinger metric on the other hand is a valid distance metric [23], which does address the limitation of *K*(*P*,*Q*) divergence [24], and in addition has attractive properties as compared to some other distance metrics such as the Chi-squared distance [25–27]. In summary, although not the only distance metric that could be pertinent for the purposes of the current work, the Hellinger distance was chosen, as it was suitable for the data at hand, had attractive properties compared to other distance metrics, and had relatively wide use in LDA context (see for example [27–30]). It should be noted in passing that the Hellinger distance is directly related to the Euclidean distance, since the Hellinger distance between vectors *P* and *Q* is equivalent to the Euclidean distance between vectors $\sqrt{P}\;and\;\sqrt{Q}$ (normalized by $1/\sqrt{2}$).

**Fig 1:**
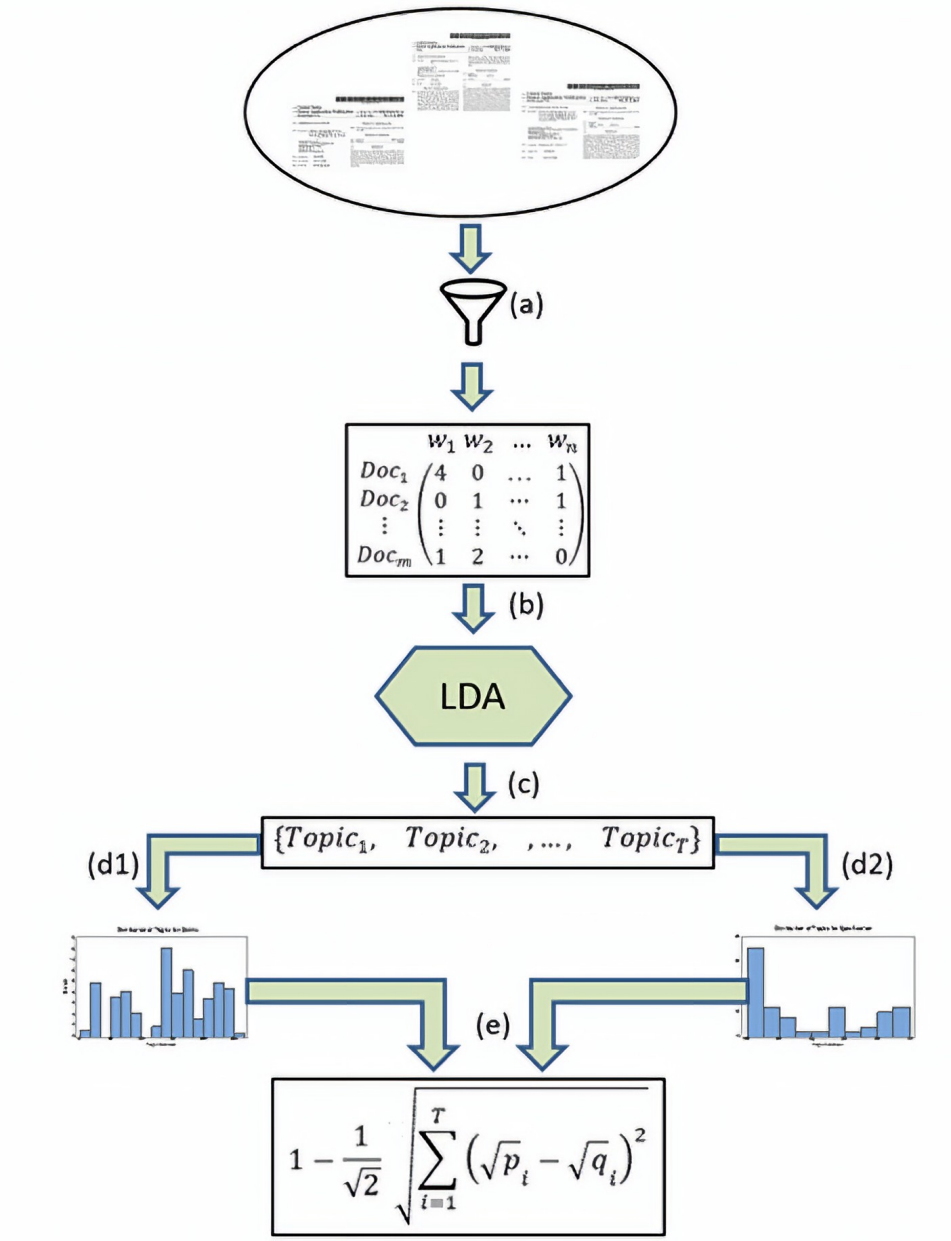
A flowchart depicting the steps taken to define drafting alignment: a) the document corpus is pre-processed to arrive at the document-term matrix; b) the result is passed to LDA; c) topics resulting from the LDA model are obtained; for each document the relative frequency distribution of the uncovered topics in d1) claims and d2) specification are obtained; e) the Hellinger distance is calculated between the resulting two distributions and drafting alignment is defined as one minus that distance.

The entire process behind the definition of drafting alignment is visualized in Fig 1. It results in each document being assigned a single number (score) between 0 and 1.

Because the novel measure of alignment is based on Hellinger distances between frequency distributions, high alignment corresponds to the claims and specification sections closely resembling each other in terms of the frequency of topics appearing in each. Low alignment on the other hand indicates that there are stark differences in topical coverage across claims and specification. Since the similarity of topical coverages of claims and specification is one important indicator of how consistent the two sections are to each other, it follows that high alignment should be preferred to low alignment. That being noted, there will often be topics that appear with different frequencies across the claims and specification sections, or that are in one section but not the other (e.g., topics that appear in the specification but not claims). That also stems from the patent law, according to which an adequate specification is expected to contain topics that are intended to explain the details behind the invention and elaborate on its uses [2]. Among those, there often will be topics that either were not used at all or were not used nearly with the same frequency in the claims section of the corresponding document. While high alignment should be preferred to low alignment, a close to perfect alignment (i.e. 1) is often neither going to be likely in practice nor satisfactory because of the patent law.

Our measure of drafting alignment addresses the two limitations implied by the alignment measure of [7]. First, instead of focusing our attention on single words (unigrams) making up each body of text (i.e. claims and specification, respectively), we identify topics making up each of the claims and specification sections. Whereas focusing on unigrams is somewhat arbitrary, as is the case with [7], working with topics alleviates that inherent subjectivity. Second, while the measure of [7] does not take into account the frequency with which unigrams appear in each body of text, our measure instead uses the relative frequency distribution of topics and is based on comparing topic distributions across the claims and the specification using Hellinger distance.

## Application and examples

### Data and pre-processing

To provide a proof of concept and illustrate our novel measure of drafting alignment, we use the documents pertaining to cybersecurity for business data processing applications. In particular, we illustrate using data specifically designed or used for cybersecurity in “the practice, administration, or management of an enterprise, or in the processing of financial data” applied to USPTO on or after November 29, 2000 (date when AIPA was enacted). Since USPC Class 705 contains patents designed or used for “the practice, administration, or management of an enterprise, or in the processing of financial data”, our data collection started by pulling all documents applied to USPTO on or after November 29, 2000 and classified into USPC main class 705. The data acquisition started with the collection of 116,288 documents. The data were acquired from two patent research databases, Questel’s *Orbit Intelligence* database and Clarivate’s *Derwent Innovation*. Note that patent data can also be obtained from publicly available sources such as USPTO’s patent search databases (https://www.uspto.gov/patents-application-process/search-patents). Patent text was acquired in XML format from the *Orbit Intelligence* database. From the XML file, we identified and used all the text populated under the <DESC> tag as the specification of a document, unless the tag was unavailable, in which case the specification was identified “manually” by locating the section with the most detailed description of the invention. Documents that were not granted at the time of data acquisition were kept in their application format, while documents that were already granted were retained in their granted format. A few initial pre-processing steps were applied to the data. Those steps included the removal of documents that had duplication by application number, in which case documents were retained in their latest version only. A similar pre-processing procedure was applied to document text, whereby documents that shared the exact same claims and specifications were retained only in their latest version. Further, documents with missing CPC classification as well as documents for which it was not possible to uniquely identify section, class, subclass, and main group of the primary CPC classification were dropped. In addition, documents that did not have a specification section were removed from the data.

Afterwards, the pre-processed business data processing patent data were filtered to arrive at the subset that directly pertained to cybersecurity. To identify those CPC classification codes that relate to cybersecurity we used the Glossary of Key Information Security Terms provided by the National Institute of Standards [31], followed by manual filtering to arrive at the cybersecurity subset. As a result, our focal dataset for cybersecurity contains 2,393 documents from the pre-processed set, which have as their primary CPC code one of our identified CPC codes (Table 1).

**Table 1:** CPC codes (up to Main Group) and titles pertaining to cybersecurity for business data processing, together with the number of documents falling under each code.

Code	Title	Number of Documents
G06F21	*Security arrangements for protecting computers*, *components thereof*, *programs or data against unauthorised activity*	1,412
H04L63	*Network architectures or network communication protocols for network security*	716
H04L9	*Cryptographic mechanisms or cryptographic arrangements for secret or secure communication*	192
H04W12	*Security arrangements*, *e*.*g*. *access security or fraud detection; Authentication*, *e*.*g*. *verifying user identity or authorisation; Protecting privacy or anonymity; Protecting confidentiality; Key management; Integrity; Mobile application security; Using identity modules; Secure pairing of devices; Context aware security; Lawful interception*	68
H04K1	*Secret communication*	5

### Topic modeling

Having arrived at the 2,393 pre-processed documents pertaining to cybersecurity for business data processing, a 360-topic LDA was applied using the *LDA* function of the R *topicmodels* package [32]. Terms that were extremely rare, i.e. occurring only once throughout the 2,393-document corpus, were removed prior to applying LDA. Note that the choice for the number of topics was motivated by a 5-fold cross-validation to test the generalization performance of LDA with various numbers of topics ranging from 2 to 500. As a quantitative tool to guide our selection number of topics, we used perplexity [33]. It should be noted that perplexity is an indicator of the *predictive* quality of a model [34], and lower values of perplexity tend to indicate a better generalization performance [13]. In automatic speech recognition, predictive perplexity measures the average uncertainty of a recognizer when predicting the *next* word based on observed history [35]. Further, from the statistical standpoint, perplexity is based on the likelihood function evaluated on data that are not used in building the model (i.e. are held out), which once again points to the predictive characteristic of perplexity. An attractive property of perplexity is that it is highly correlated with the word error rate (WER), a measure of performance in automatic speech recognition. This implies that minimization of perplexity is a meaningful goal in language modeling [35]. In topic models, perplexity has been widely used to gage predictive, or equivalently, the generalization performance (see for example [13], [16], [28], [29], [36]).

It should be noted that perplexity is not the only measure that can be used for topic number selection. Measures of topic coherence [37] in general, and UCI or UMASS metrics in particular ([38] and [39], respectively), have found use in recent years when the emphasis is on improving the interpretability, or equivalently, the descriptive characteristics of topics resulting from a model. We adopted predictive perplexity rather than descriptive coherence in this work for two main reasons. First, it is the predictive quality of the topic model that is of pertinence when, for example, an inventor or patent examiner needs to quantify the alignment (based on results from a topic model) of a *new* application or a patent based on *historic* patent data. Second, albeit fundamental for the calculation of drafting alignment, in the current work, topics themselves are only means to an end–the end being the calculation of alignment using the results from the topic model. As such, from a practical standpoint, the exploratory (descriptive) investigation of topics is of lesser importance than the calculation of alignment.

**Fig 2:**
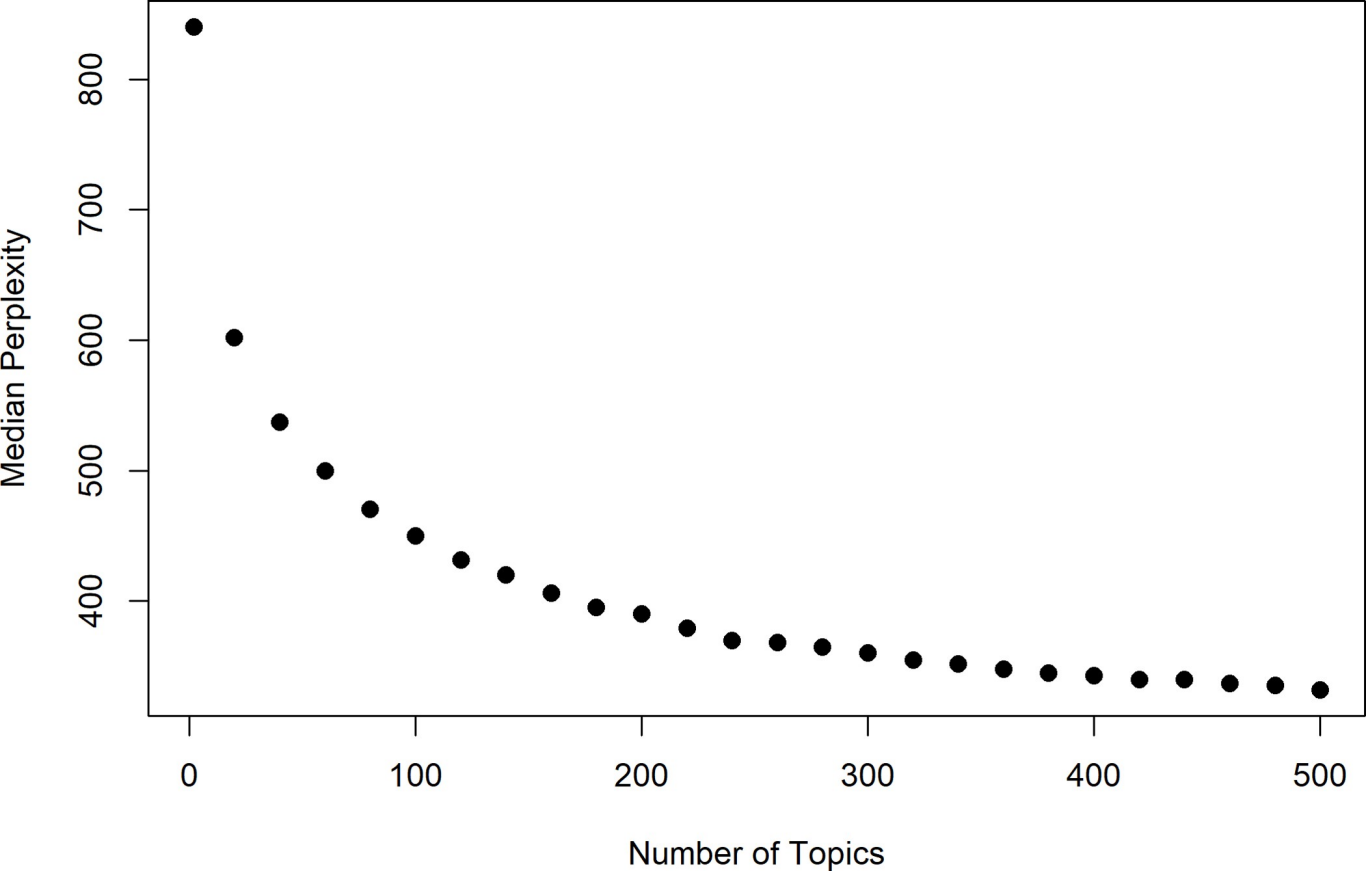
Median perplexity vs number of topics based on the results of 5-fold cross-validation to determine a suitable number of topics for LDA. Each dot corresponds to the median of perplexities over the five folds run for the respective topic.

For each contingent number of topics, perplexities emerging from different iterations of the cross-validation were calculated and stored. For a validation set consisting of a collection of documents, perplexity is defined as

$$Perplexity=e^{-\frac{log(p(w))}{N}}$$

where **w** is the collection of documents from the validation set, log(*p*(**w**)) is the log-likelihood, and *N* is the aggregate number of terms in the documents of the validation set. Lower values of perplexity are indicative of a better generalization performance of a topic model [13]. Fig 2 shows the scatterplot of median perplexity versus the different number of contingent topics tested. Based on these results, the number of topics where the median perplexity starts to plateau was visually identified at about 360 topics.

Although due to space considerations each of the 360 topics is infeasible to present in the current manuscript, provided in Table 2 are the results for a few of the notable topics emerging from the application of 360-topic LDA. Of note, for each of the provided topics, in Table 2 we present only the top three terms that have the highest conditional probability $\phi_{w}^{\left(j\right)}$, in other words, terms that for the given topic are the most “representative.” Note that terms appearing in the table are stemmed based on Porter’s algorithm [18]. An exemplary topic, topic III pertains to sensor technologies for wearable devices. A patent application containing that topic with high probability is “Method and apparatus for off-body detection for wearable device” (US20160154952A1), originally assigned to Fitbit, Inc., and intended for detecting, based on input from biometric sensors, when a wearable technology is removed from the user. Another example is topic IV related to digital rights management, and a patent application containing this topic with a high probability is “Specifying rights in a digital rights license according to events” (US20020019814A1), originally assigned to Microsoft Corporation. The invention relates to architectures that allow access to encrypted digital content in line with rights specified in licenses.

**Table 2:** Results for selected “topics” from an LDA implementation for cybersecurity documents for business data processing.

Topics				
I	II	III	IV	V
Event	Profil	Sensor	Licens	Target
Fraud	Network	Devic	Content	Privaci
Detect	Social	Wearabl	Digit	Relationship

For each “topic”, only terms with the highest conditional probability are displayed. Note that terms appearing in the table are stemmed. The enumeration of topics using Roman numerals is arbitrary.

### Examples

**Fig 3:**
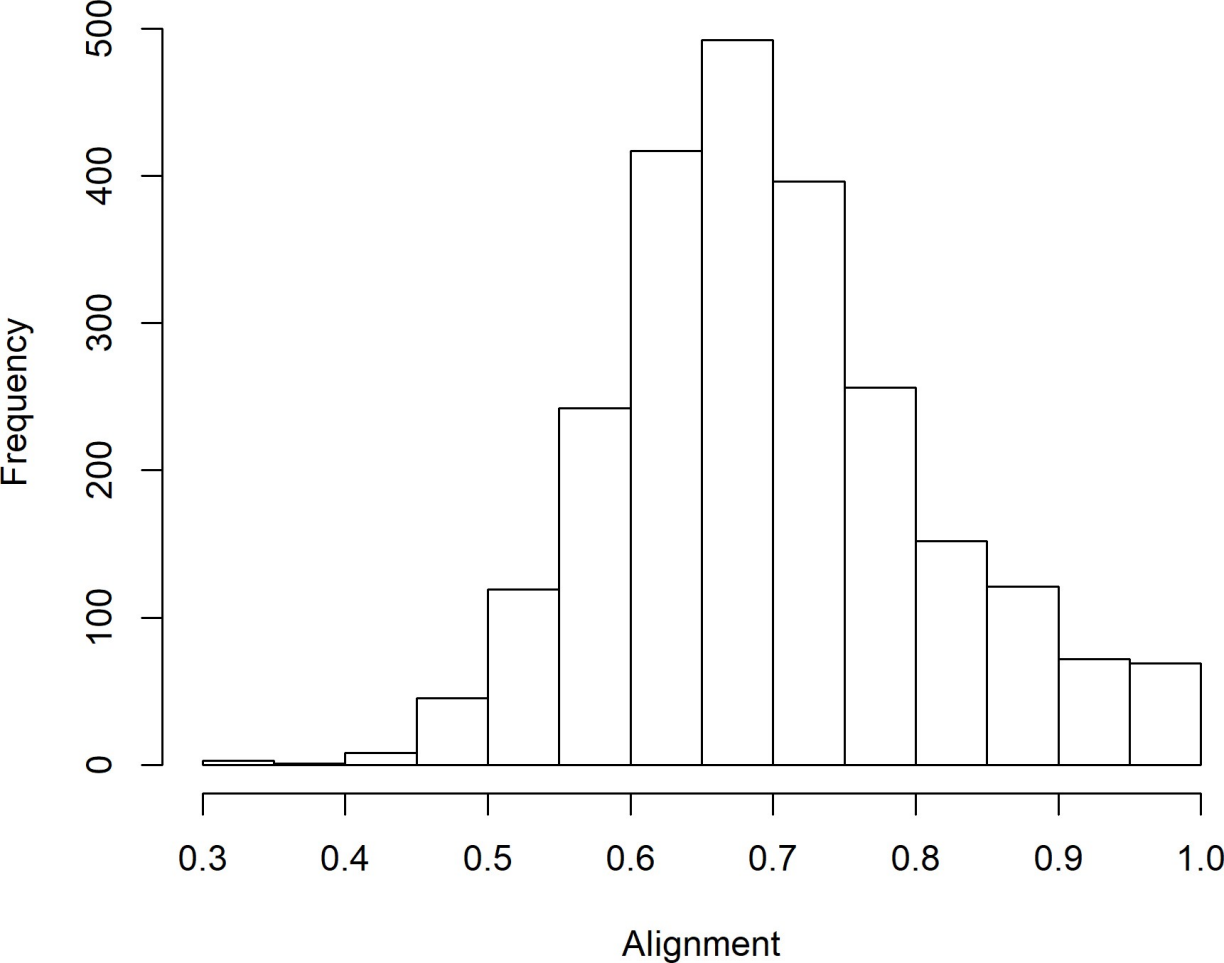
Histogram of drafting alignment for the 2,393 documents pertaining to cybersecurity for business data processing.

For each of the 2,393 documents, the relative frequency distribution of the 360-topics was obtained across the respective claims and specification sections. In other words, for each of the two bodies of texts, after every word was assigned to the most likely topic, the relative frequency distribution of topics was determined within the body of text. Subsequently, the Hellinger distance was calculated between those pairs of distributions and drafting alignment defined as one minus the Hellinger distance. As a result, each document was assigned a drafting alignment score that ranged between 0 and 1. Fig 3 displays the histogram of the resulting 2,393 alignments. The distribution is symmetric, with a mean of 0.70, a median of 0.69, and a standard deviation of 0.11.

**Fig 4:**
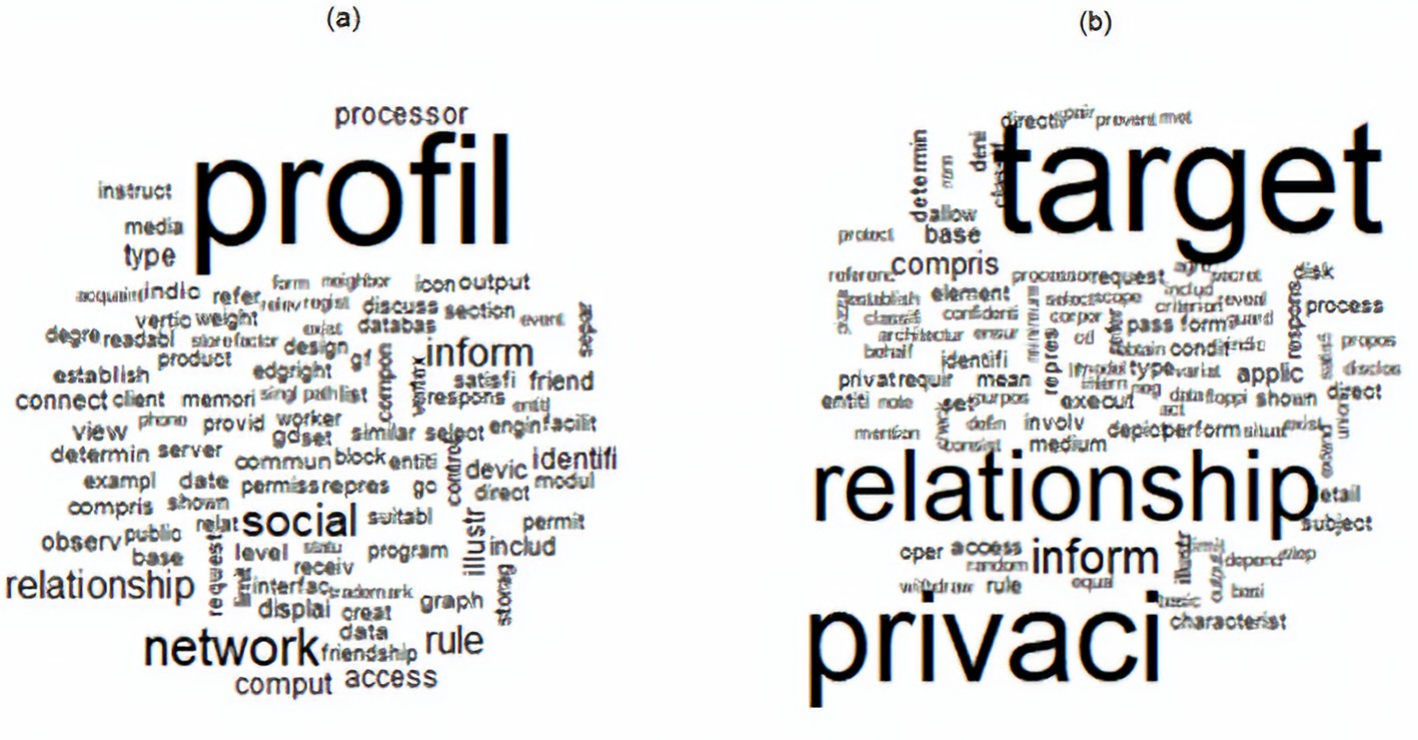
Wordclouds of topics II (a) and V (b) for patent US7716140B1. The figure displays only the top 100 terms (stemmed) within each topic when ranked according to probability $\phi_{w}^{\left(j\right)}$. Note that for each topic the terms are sized based on the magnitude of probabilities relative to the other terms within the topic.

As an example of a document with a relatively high alignment, consider patent number US7716140B1 titled “Methods and Systems for Controlling Access to Relationship Information in a Social Network” granted to Google, Inc., on May 11, 2010. The patent pertains to accessibility of relationship information existing between members in a social network. The patent describes how to control the access of information based on privacy rules that need to be satisfied when an observer requests to receive relationship information on another member. There are two topics with non-zero probabilities making up the specification section. Those topic numbers, with the corresponding probabilities (Q) are topic II (see Table 1) with a probability 0.83 and topic V (see Table 1) with a probability 0.17. In fact, those two topics are the only topics that feature also in the claims section of the patent with non-zero probabilities (P). In particular, topics II and V feature in claims with probabilities 0.78 and 0.22, respectively. The wordcloud for those two topics is depicted in Fig 4.

As can be expected from the dominant presence of topic II in both claims and specification, the drafting alignment calculated based on the method outlined above is relatively high, equaling

$$1-\frac{1}{\sqrt{2}}\sqrt{{\left(0.78-0.83\right)}^{2}+{\left(0.22-0.17\right)}^{2}}\approx 0.95$$

As an illustration of a document that is not well-aligned, note the abandoned patent application US20030233328A1, titled “Method and system for securely communicating data in a communications network” assigned to PATENTEK, Inc. Based on the claims, the application pertains to two devices communicating with each other through an encrypted communication session and each comprising of a processor and a memory, the latter storing encryption and decryption processes that are being used for the encrypted communication. Both the claims and the specification sections of this document have the same topic appearing as the most dominant which, unsurprisingly, relates to memory and storage. However, the second most dominant topic in the specification section pertains to servers as well as receiving and transmitting of information, whereas that topic does not feature in the claims section at all. Instead, the second most “important” topic in the claims relates to cryptography, and that topic is only the fifth most dominant topic in the specification section. This abandoned application has an alignment of 0.40.

**Fig 5:**
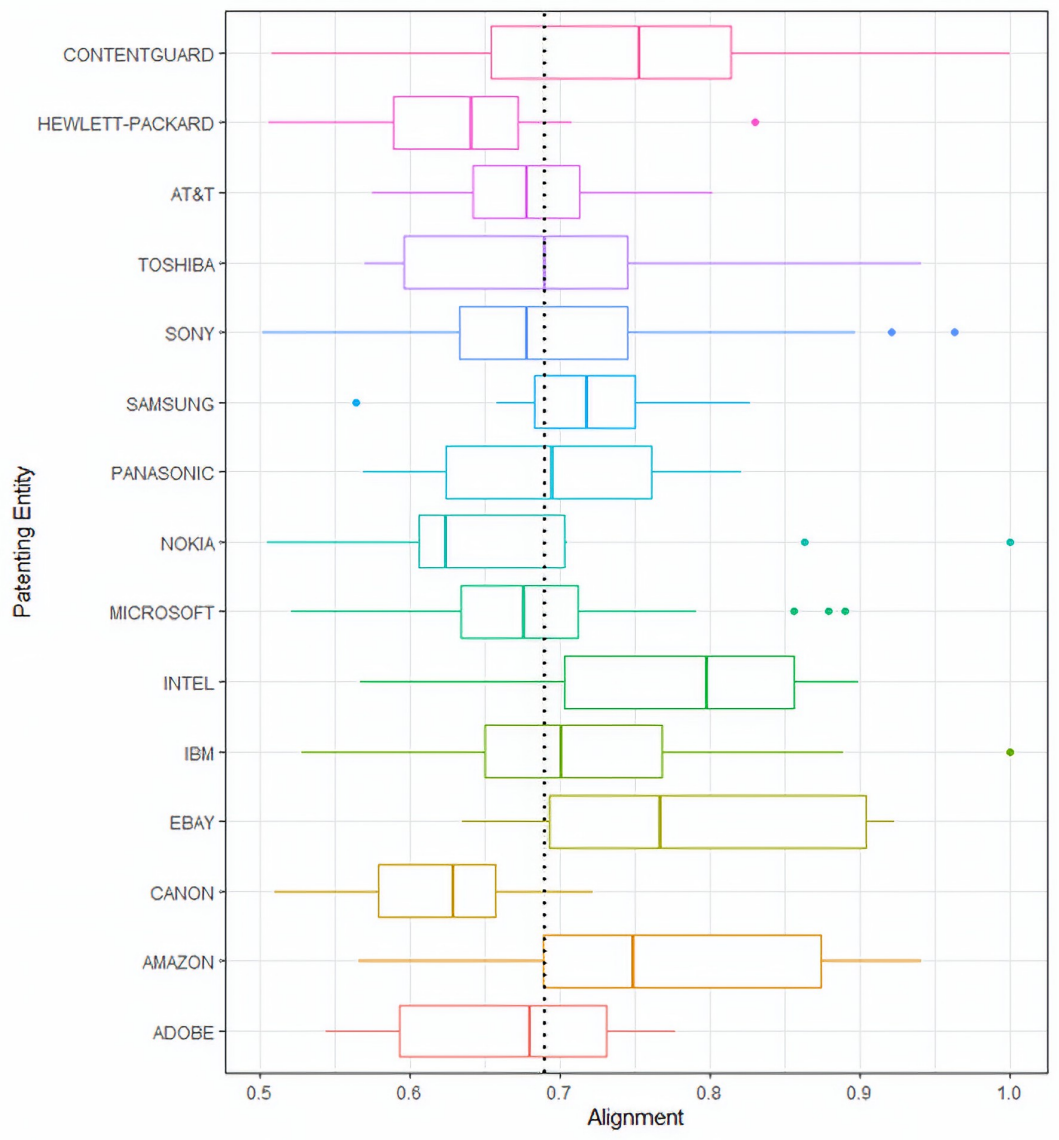
Drafting alignment distributions for patents of top assignees with at least 10 granted patents in the focal data. The vertical dotted line corresponds to the median alignment among all granted patents in this domain.

Displayed in Fig 5 are boxplots, showing distributions of drafting alignment for (granted) patents of top assignees, i.e. assignees that had at least 10 granted patents in our focal data. The vertical dotted line corresponds to the median alignment of all granted patents in the focal data (0.69). Information displayed in Fig 5 can be used by the organizations that hold patents, for example, to understand where the organization stands in terms of the alignment “profile” of its patent portfolio. For instance, an organization such as ContentGuard Holdings, Inc. could learn from such an analysis that the median alignment of their patents is well over the domain-specific median (0.69). Similar analysis can be helpful for investors (e.g. venture capitalist) who, before investing in a firm, might want to safeguard themselves from the risk of losing and otherwise valid patent due to poor alignment, in future litigation. For this reason, they may want to know the alignment distribution of the firm’s patents as part of their due diligence. If the alignments of granted patents of the focal firm in question are not well-aligned then, if making the investment, the investor might be at a risk of future litigation in case some of those poorly aligned patents are challenged for validity.

**Fig 6:**
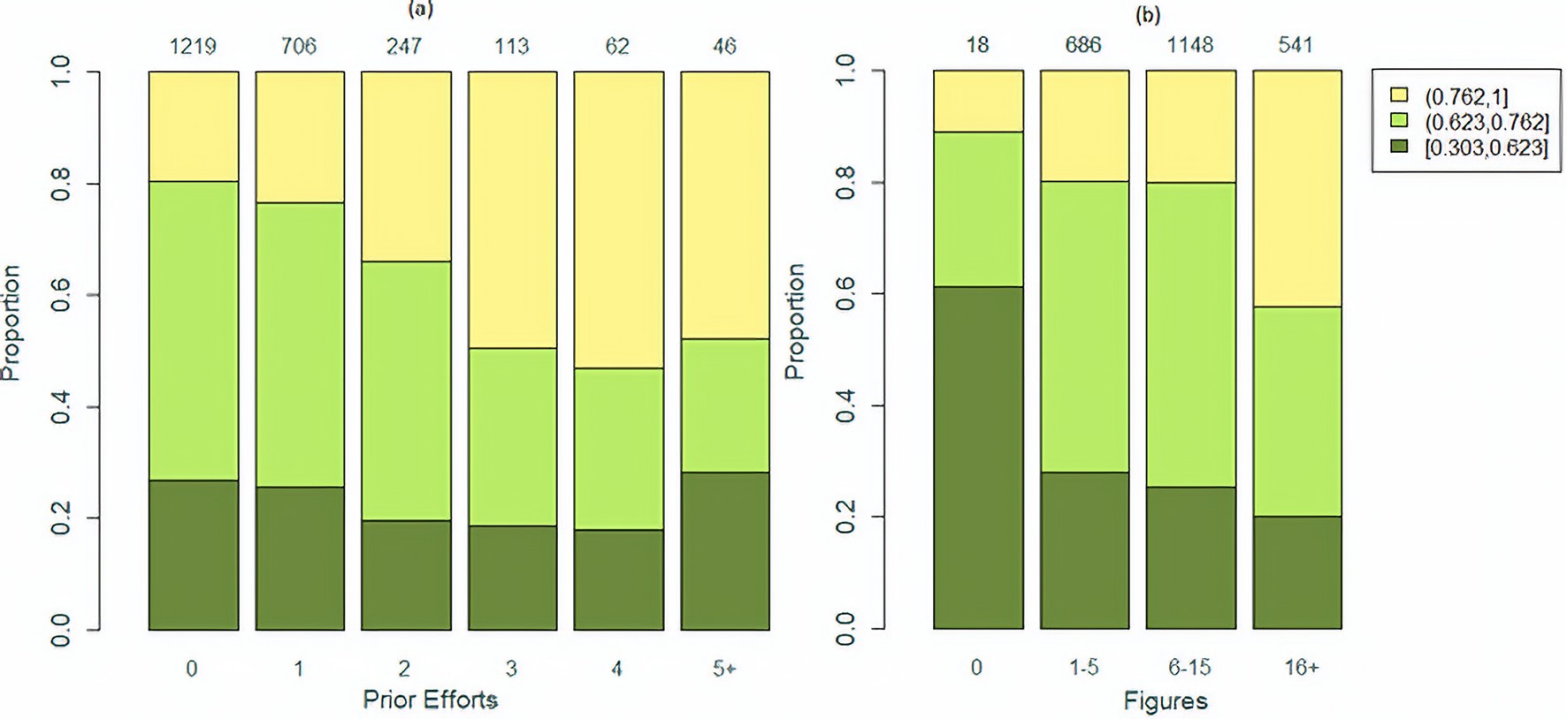
Levels of alignment of business data processing patent documents for cybersecurity across different levels of (a) prior efforts and (b) number of figures displayed in a document. The dark green color corresponds to documents with “low” alignments, the light green color corresponds to documents with “typical” alignments, while the yellow color corresponds to documents with “high” alignments. The numbers appearing on top of each bar correspond to the total number of documents corresponding to the bar.

In Fig 6(A), we display the relationship between alignment and the number of prior efforts. We define prior efforts as the number of non-provisional applications that are related to the document as its “predecessors.” For instance, non-provisional applications to which the document under consideration claims priority as being their continuation, continuation in part or divisional (and all documents to which those in turn claim priority, ad infinitum) qualify as a prior effort. This field serves as a proxy for attempts that were made at patenting the given invention or closely related inventions. We define prior efforts using the *Related Applications* field obtained from *Clarivate’*s Derwent Innovation patent database. For the purpose of Fig 6(A) we converted the resultant discrete variable to categorical format by keeping levels 0–4 as they are, and grouping all else under the level 5+. In Fig 6(A), for each level of the newly defined variable we show the proportion of documents having low, typical, and high alignments, where “low” refers to alignment below the first quartile, “typical” refers to alignment between first and third quartiles, and “high” refers to alignment above the third quartile. As can be seen from the figure, there is an increasing trend in the proportion of highly aligned documents as the prior efforts increase up until the number of prior efforts reaches four. Conversely, the proportion of documents that are not highly aligned generally goes down with the increase in prior efforts, again up until four prior efforts. An associated significance test for Kendall rank correlation [40] results in a rank correlation of 0.123 with an associated p-value of less than 0.0001.

A similar pattern can be observed when alignment is investigated in relation to the number of figures used in a document, as illustrated in Fig 6(B). It can be noticed, that the lowest proportion of highly aligned documents occurs for those documents that have no figures. For documents that use no more than 15 figures (75^th^ percentile of the distribution for the number of figures), the proportion of documents with high alignment is about twice as high as that of the documents with 0 figures. Finally, for the documents that make extensive use of figures by employing more than 15 figures the proportion of highly aligned documents is the highest. The related Kendall rank correlation [40] for these data is 0.137 with a p-value of less than 0.0001. This finding resonates with recent arguments that incorporation of multiple figures can be extremely useful when describing the invention [41].

## Drafting alignment and USPTO actions

Finally, yet importantly, we turn to understanding the relationship between the novel measure and the adherence to the written description requirement in the patent law [2]. Put differently, is the novel measure defined in this article in agreement with USPTO actions in relation to the written description requirement outlined as part of §112 of Title 35 of USC? To that end, we use all the pertinent data from the research datasets made publicly available by the USPTO. We used the *Office Action Research Dataset for Patents* [42], in particular the two tables related to office actions and rejections.

Note that those data pertain to all USPTO office actions that were mailed to inventors starting from the middle of 2008 and ending around the middle of 2017. Given the absence of pre-mid-2008 data, we only focused on the subset of our focal data that were filed to the USPTO on or after January 19, 2006. Based on the patent accountability report for the fiscal year 2007 [43], the average first action pendency time was reported as 25.3 months (overall) and 25.9 months for the technology center responsible for the vast majority of rejections related to the written description requirement of §112 (a), Title 35 of USC in our focal data. Based on those statistics we proceeded with the investigation using only the subset of our focal data that corresponded to applications filed with the USPTO no earlier than 26 months from the earliest rejection marked in the office actions table. That resulted in excluding from investigation all the documents that were filed with the USPTO before January 19, 2006. Further, we only considered documents that were not yet granted. We found 120 rejections directly related to the written description requirement. Each of those rejections meant, that it had been determined during the examination at the USPTO that the patent application had not been meeting the written description requirement. The survival time of each such application was equal to the amount of time from its filing to when the letter was mailed by the USPTO notifying, among other things, of the lack of meeting the written description requirement. The survival times of the remaining applications were right censored, as the failure to meet the written description requirement was not established for those applications.

**Fig 7:**
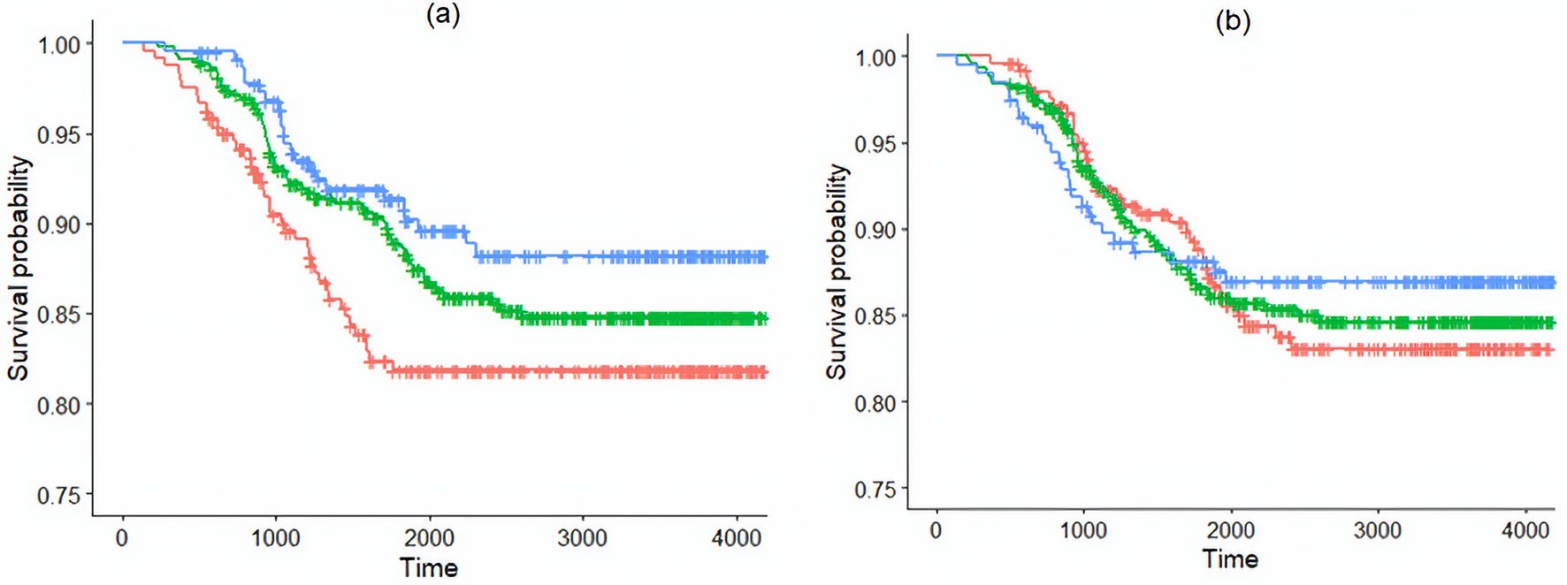
Estimated survival functions for the three groups of patent applications with “high” (blue curve), “typical” (green curve), and “low” (red curve) alignment for (a) the novel definition of drafting alignment, and (b) if alignment is defined in line with [7].

To answer our question of whether the novel measure is in agreement with the written description requirement for documents that were not granted, we plotted the estimated survival functions using the Kaplan-Meier (product-limit) estimator [44] for each of the three groups of documents having respectively “low,” “typical,” and “high” alignment (as defined earlier). As can be seen from Fig 7(A), for any fixed time the estimated survival probability is the highest for the documents falling into the group that are highly aligned, followed by the groups with a typical alignment and low alignment, respectively. Thus, from this investigation, using the subset of our data, we conclude that the estimated probability of not meeting the written description requirement is the highest for applications with low alignment (red curve) and lowest for applications with high alignment (blue curve). What that implies for the subset of data considered for this analysis, is that the novel measure of drafting alignment is generally in line with the office actions at the USPTO. The associated significance tests for the comparison of survival curves result in p-values of 0.098 for the log-rank and 0.078 for the Peto and Peto’s (1972) test [45], [46]. The somewhat inconclusive magnitude of the p-values may be partly explained by the relatively small sample of §112 (a)-related rejections, as only 120 documents were rejected for reasons associated with the requirement of §112 (a). Note, that had the alignment been defined in line with [7], where instead of comparing topical coverages across claims and specification sections, only the presence or absence of individual words was considered (in each of claims and specification sections), we would have obtained the curves presented in Fig 7(B). As can be seen, there is no clear separation among the curves corresponding to low, medium, and high alignments, and that resonates with the corresponding p-values for testing the difference in survival curves, which are 0.79 for the log-rank test and 0.87 for the Peto and Peto’s (1972) test [45], [46].

Although not the purpose of this article, the predictive potential of the proposed measure of alignment is worthy of a consideration. To understand whether, if at all, the drafting alignment can be useful as a predictor for classifying the status of an application we tentatively entertained the Cox proportional hazards model [47], with the drafting alignment as the only predictor. The binary classification considered in our model attempted to differentiate between an application being rejected for reasons related to §112 (a) vs. not being rejected related to §112 (a). The data used for this tentative model comprised all (non-granted) patent applications that were used in the discussion above (Fig 7). We performed 10-fold cross-validation, recording the area under the Receiver Operating Characteristic (ROC) curve for each of the 10 iterations. The area under the curve, averaged across the 10 iterations was 0.74, which is indicative of “acceptable” discrimination [48].

## Conclusion

The written description requirement is an easily overlooked requirement for obtaining a patent and for keeping it. An inadequate written description to elucidate the claims can lead to delays or the ultimate rejection of a patent application during prosecution. Once a patent is issued, it can be challenged for its validity and litigated, for instance by an alleged infringer, which can result in costly litigation for the patent owner and potential death of the patent. Notable numbers of invalidations result from a lack of alignment between claims and specification [7].

The novel measure of drafting alignment that we propose can guide inventors and their agents to improve the written descriptions of the applications they submit, as well as enable patent examiners to streamline patent prosecution by separating well-aligned documents from those that are less coherent, similar to recommendations of [7]. The measure proposed herein can easily be automated to allow the preparers of patent applications to self-check their descriptions. It may also serve to notify the examiner of a poorly aligned application. Patent offices can also establish benchmarks based on data from well-aligned patent descriptions. Incoming patent applications can then be compared with those benchmarks and returned to inventors for improvement if they are significantly falling short of those benchmarks. The proposed measure can also be adopted into a web app/interface and required during patent submission. Inventors whose applications fall short of a certain threshold (e.g. first quartile or median) of alignment may be directed to improve the alignment before the submission. The web interface could also notify the inventor of the particular areas where the application is aligned poorly and needs most improvement. Having such information, an entrepreneur or inventor can be alerted of crucial pitfalls that might be lurking in the future, if an application with low alignment were submitted as is.

The measure proposed herein could also be useful for investors. It is well known, that having patents on their inventions helps entrepreneurs secure capital for their ventures [49]. To exercise the patent rights, however, the entrepreneur has to have a valid patent. Thus, if a patent is to lose its validity in the future then it is not going to deliver the promise that investors hoped for when investing in the venture. Since alignment and validity are directly related, it would be useful for investors to have means of quantifying the alignment of patents in question and knowing when patents are poorly aligned and at a high risk of later being invalidated. The measure of drafting alignment proposed herein serves as a convenient tool enabling investors to avoid investing in technologies, which are patented, but at a risk of future invalidation due to insufficient alignment of the description with the claims.

A few limitations of this work are worthy of special note. One limitation arises because of an assumption inherent in LDA. In particular, LDA is a “bag of words” model that operates on unigrams. It follows that certain combinations of unigrams that conceptually should be part of a given topic are in fact allowed to feature in other topics as well [13]. It has been suggested that this limitation could be overcome in practice by relaxing the assumption that topics are made of unigrams, and instead extending LDA for use with expressions made up of n-grams [13]. Another limitation is due to the way patents are classified. Occasionally IPC/CPC schemes are supplemented with new classifications corresponding to recent technologies. The novel measure proposed in this article presupposes the existence of adequate data on which LDA can be applied and topics can be “learned.” If the domain in which drafting alignment is sought is emerging and patents in that field are scarce, then the corpus and hence the vocabulary based on which LDA uncovers the topics might be insufficient. Third, our attempt to understand the consistency of the proposed measure with the USPTO actions was based on the data that were available to us through the open access *Office Action Research Dataset for Patents*, as pertaining to office actions and rejections. That data, as it was mentioned earlier, only covered part of our focal data set in terms of when the notices were mailed to inventors. Because the pre-mid-2008 data were missing from the openly accessible dataset, we were able to focus only on the subset of our focal data. Fourth, the current work did not address synonymy as part of pre-processing. Consequently, the resulting topics and thus the drafting alignment did not account for the fact that two different words appearing in claims and specification respectively, might carry a close or even the same meaning, as is the case with synonyms.

One area of future research is an in-depth investigation, using a more comprehensive data, of the extent to which examiner decisions agree with drafting alignment. Another area worthy of future investigation is the potential association of drafting alignment with factors not considered in the given work. Such factors include but are not limited to the patent examiner working on the application at the USPTO, the attorney or agent assisting the applicant with filing for a patent, and the art unit responsible for the examination at the USPTO. Another interesting extension would be a rigorous treatment for synonymy. The latter may be achieved by incorporating in data pre-processing a similarity measure between word vectors stemming from a “Word2vec” framework [50], [51], followed by standardization of words with the similarity measures exceeding a pre-defined threshold. Alternatively, assuming the presence of a fixed, domain-specific database of synonyms, one could standardize the synonyms based on their appearance in the noted database, as part of data pre-processing. A summary of further approaches for synonym identification can be found in [50].

In closing, we note that we focused on a subset of business data processing documents for cybersecurity to illustrate the proposed measure. However, the main contribution of the paper–the statistical approach for the definition of alignment–is invariant of the application domain and thus adaptable to other domains, given the presence of adequate data. Indeed, the proposed definition of alignment and the steps taken to calculate it (LDA, Hellinger distance, etc.) would be relevant regardless of the domain where the alignment is sought. The approach to defining alignment is thus robust and can readily be applied to other domains.
